# Searching for new antifungals for the treatment of cryptococcosis

**DOI:** 10.1590/0037-8682-0121-2023

**Published:** 2023-07-24

**Authors:** Naira Sulany Oliveira de Sousa, Juan Diego Ribeiro de Almeida, Hagen Frickmann, Marcus Vinícius Guimarães Lacerda, João Vicente Braga de Souza

**Affiliations:** 1 Programa de Pós-Graduação em Biodiversidade e Biotecnologia da Rede BIONORTE, Manaus, AM, Brasil. Programa de Pós-Graduação em Biodiversidade e Biotecnologia Rede BIONORTE Manaus AM Brasil; 2 Instituto Nacional de Pesquisas da Amazônia, Manaus, AM, Brasil. Instituto Nacional de Pesquisas da Amazônia Manaus AM Brasil; 3 Institute for Medical Microbiology, Virology and Hygiene, University Medicine Rostock, Germany. Institute for Medical Microbiology, Virology and Hygiene University Medicine Rostock Germany; 4 Department of Microbiology and Hospital Hygiene, Bundeswehr Hospital Hamburg, Germany. Department of Microbiology and Hospital Hygiene Bundeswehr Hospital Hamburg Germany; 5 Fundação de Medicina Tropical Dr. Heitor Vieira Dourado, Manaus, AM, Brasil. Fundação de Medicina Tropical Dr. Heitor Vieira Dourado Manaus AM Brasil; 6 Instituto de Pesquisas Leônidas & Maria Deane, Fiocruz, Manaus, AM, Brasil. Instituto de Pesquisas Leônidas & Maria Deane Fiocruz Manaus AM Brasil; 7 University of Texas Medical Branch, Galveston, USA. University of Texas Medical Branch Galveston USA

**Keywords:** Cryptococcosis, Therapeutic failures, Anticryptococcal drug development

## Abstract

There is a consensus that the antifungal repertoire for the treatment of cryptococcal infections is limited. Standard treatment involves the administration of an antifungal drug derived from natural sources (i.e., amphotericin B) and two other drugs developed synthetically (i.e., flucytosine and fluconazole). Despite treatment, the mortality rates associated with fungal cryptococcosis are high. Amphotericin B and flucytosine are toxic, require intravenous administration, and are usually unavailable in low-income countries because of their high cost. However, fluconazole is cost-effective, widely available, and harmless with regard to its side effects. However, fluconazole is a fungistatic agent that has contributed considerably to the increase in fungal resistance and frequent relapses in patients with cryptococcal meningitis. Therefore, there is an unquestionable need to identify new alternatives or adjuvants to conventional drugs for the treatment of cryptococcosis. A potential antifungal agent should be able to kill cryptococci and “bypass” the virulence mechanism of the yeast. Furthermore, it should have fungicidal action, low toxicity, high selectivity, easily penetrate the central nervous system, and widely available. In this review, we describe cryptococcosis, its conventional therapy, and failures arising from the use of drugs traditionally considered to be the reference standard. Additionally, we present the approaches used for the discovery of new drugs to counteract cryptococcosis, ranging from the conventional screening of natural products to the inclusion of structural modifications to optimize anticryptococcal activity, as well as drug repositioning and combined therapies.

## INTRODUCTION

Cryptococcosis, a potentially fatal fungal infection in immunosuppressed patients, especially in those infected with human immunodeficiency virus (HIV), is caused by the inhalation of encapsulated yeasts belonging to the *Cryptococcus neoformans and Cryptococcus gattii species complex*^1^. It is associated with high mortality in low- and middle-income countries, and causes approximately 181,000 deaths annually^2^^-^^3^. Sub-Saharan Africa reports the highest number of cases, with approximately 720,000 cases per year, followed by Southeast Asia and Latin America, which are the second and third regions most affected by cryptococcal meningitis^3^^-^^4^. 

Results of antifungal therapies for cryptococcosis are limited. Depending on an individual’s immune status, disease severity, and availability of antifungals, the standard treatment is based only on amphotericin B, fluconazole, and flucytosine^5^^-^^6^. Owing to its relatively low cost, high oral bioavailability, and low toxicity profile, fluconazole is often used to replace amphotericin B and flucytosine in resource-limited settings. However, resistant fungi and persistent therapeutic failure have been observed in patients with cryptococcosis undergoing prolonged therapy with fluconazole^7^. In addition, the limited antifungal arsenal, serious adverse effects of amphotericin B and flucytosine, and intrinsic resistance of *C. neoformans* to echinocandins, the only new broadly available class of tantifungal drugs developed in decades, have stimulated new studies in search of better antifungal agents to treat cryptococcosis^8^^-^^10^. 

Drugs can be discovered in natural products that, since antiquity, have been an important source of attractive bioactive compounds for drug development or can be produced through full or partial synthesis^11^. However, despite advances in molecular techniques and medicinal chemistry, the development of new drugs remains slow and expensive. In addition, several drug candidates are barred during the transition from the preclinical to the clinical stage, with 89% failing due to toxicity^12^. Thus, the reuse of drugs, that is, the definition of new therapeutic indications for substances already approved by the Food and Drug Administration, has attracted considerable attention. Another used approach is combining antifungal agents with other drugs, thus improving the activity of traditional antifungals due to their associated action on more than one target^10^. 

This review aims to provide an overview of the scientific evidence available for cryptococcosis in general, current treatment options, therapeutic failures, and methodologies for obtaining new anticryptococcal drugs, for example, by bioprospecting natural products and structural modifications. In addition, it aims to address potential drugs, or drug combinations, which are undergoing preclinical and clinical investigations for drug repurposing and combined therapy.

## CRYPTOCOCCOSIS

**FIGURE 1:**
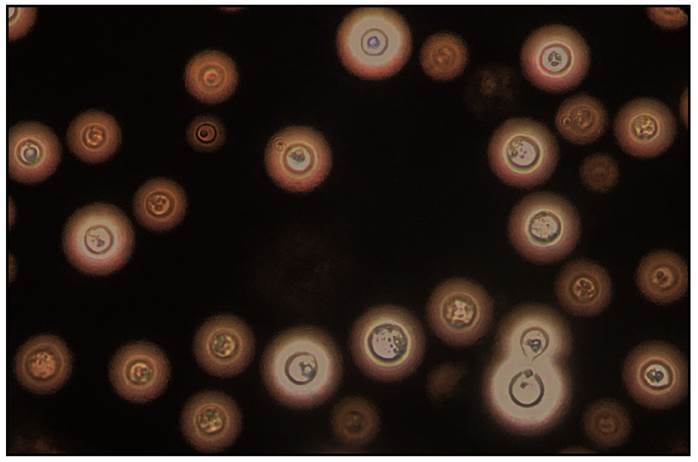
Micromorphological characteristic of *Cryptococcus* spp. Direct exam, prepared with Indian ink (400×).

Cryptococcosis or cryptococcal infection is a life-threatening fungal disease caused by the inhalation of encapsulated yeasts (Figure 1) belonging to the *C. neoformans* and *C. gattii* species complex^1^^-^^13^. With the evolution of molecular biology techniques and the use of different genotyping methods, it has become possible to assign these species to eight main genotypes: VNI, VNII, VNIII, and VNIV for *C. neoformans* and VGI, VGII, VGIII, and VGIV for *C. gatti*^14^^-^^17^. Recently, a fifth *genotype (VGV) has been described in the C. gattii* species complex^18^*.*

The causative agent is widely distributed in the natural environment, commonly in feces and birds nest, but mainly in pigeons, dead organic matter, bark, leaves, and fruit trees^17^. *Cryptococcus* spp. are globally distributed, and until 1955, prior to the availability of antifungals especially amphotericin, cryptococcosis was inevitably fatal^19^. Today, mortality remains high, particularly in the endemic regions of sub-Saharan Africa, a setting where access to healthcare is limited and the number of HIV infected individuals is high^20^^-^^21^. In developed countries, the observed drop in mortality rate can be explained by early diagnosis and wide availability of antiretroviral therapy^22^. 

Cryptococcosis occurs predominantly in immunocompromised patients and is a major cause of morbidity and mortality in these individuals, especially in those infected with HIV^21^^-^^23^. Individuals with diabetes and lupus erythematosus, transplant recipients, patients using immunosuppressive therapies, and patients with malignant neoplasms are also frequently affected with cryptococcosis, thus becoming a worldwide concern^5^^-^^7^. Cryptococcal infection also manifests in immunocompetent patients, and the signs and symptoms of infection are often nonspecific. This lack of specificity often leads to a delay in diagnosis and initiation of appropriate treatment, which in turn may lead to a severe clinical course and rapid death, even in patients without HIV^24^. In addition, delayed diagnosis can lead to additional morbidities such as stroke, blindness, deafness, neurological impairment, and cognitive dysfunction^25^. 

The primary manifestation, pulmonary cryptococcosis, can range from mild colonization of the lungs to severe lung infection^5^^-^^6^. At this stage, yeast can be spontaneously eliminated or remain in a non-replicative state for months or even years in immunocompetent hosts^26^^-^^27^. However, in cases of impaired immunity, yeasts are reactivated and disseminated via the blood to various organs, especially the brain and meninges, leading to cryptococcal meningitis. The latter is the most common and severe clinical manifestation of cryptococcosis, primarily affecting immunosuppressed patients, particularly those with depleted or defective CD4+ T cells^5^^-^^25^^-^^28^. The infection also involves other sites such as the skin, skeletal system, digestive tract, and prostate; though uncommon this is well-documented in the literature ^18^^-^^29^^-^^30^. 

## CONVENTIONAL THERAPY

Depending on the individual’s immune status, site of infection, disease severity and drug availability, several therapeutic regimens can be considered for the treatment of cryptococcosis^5^^-^^28^^-^^31^. Although adapted to the infection severity and state of the host’s immunity, the World Health Organization (WHO) recommends the treatment of cryptococcal infections using a three-stage therapeutic strategy: induction, consolidation, and maintenance. The standard therapy is limited to the use of the following drugs: amphotericin B, flucytosine, and fluconazole^28^. In summary, amphotericin B, alone or in combination with flucytosine, is employed as an initial induction therapy, and fluconazole is suggested for the consolidation and maintenance therapy^28^^-^^32^^-^^33^. 

Among the three drugs available, amphotericin B is the oldest antifungal drug for systemic use. It acts by binding to ergosterol in fungal cell membranes, forming pores that allow the leakage of cell contents, such as K^+^, Na^+^, H^+^, and Cl^−^ ions, which consecutively leads to apoptosis^34^^-^^35^. Despite being considered as one of the systemic antifungals with the broadest fungicidal activity, the use of amphotericin B has some limitations that are mainly associated with its nephrotoxicity^36^. Lipid formulations of amphotericin B with reduced toxicity have been developed; however, although liposomal amphotericin B has an improved safety profile and greater efficacy than conventional amphotericin B^7^, the cost of these lipid formulations continues to be a barrier for the treatment of cryptococcosis in resource-limited countries^37^. 

The synthetic drug flucytosine, which was first evaluated as an antitumor agent^38^, is recommended by WHO; however, it is mainly available in resource-rich countries. The drug is efficient for the treatment of cryptococcosis when combined with amphotericin B ^39^^-^^40^. However, its use as a single antifungal agent is discouraged owing to its significant adverse effects, in particular, hepatotoxicity, myelotoxicity, and resistance when used in monotherapy, thereby compromising therapeutic success^8^^-^^41^^-^^43^. 

Fluconazole is one of the best-known antifungal drugs for the systemic treatment of a broad spectrum of fungal infections. Azoles constitute a class of synthetic antifungals with fungistatic activity, and fluconazole, in particular, has been in clinical use since the 1980s^44^. In cryptococcosis therapy, the main advantage of fluconazole is its lack of severe nephrotoxic effects. Furthermore, they are frequently used to replace amphotericin B or flucytosine when their availability is limited^33^. However, because the duration of therapy is long, significant resistance is often reported in this antifungal class^7^. 

WHO has recently published new strategies and guidelines for the management of patients with cryptococcosis^28^. These protocols were established in association with a clinical trial carried out by Jarvis and colleagues^31^ that recommend the use of liposomal amphotericin B as a first-line treatment for cryptococcal meningitis. It was administered as a single dose on day one, followed by 14 days of flucytosine and fluconazole administration. The study revealed that this treatment scheme considerably improved survival rates, reduced neurological impairment, and decreased adverse events in patients with infection. The WHO stresses the importance of early diagnosis and treatment of cryptococcosis, together with recommendations of closely monitoring patients during and after treatment to avoid relapses.

In summary, access to only the antifungal drugs available for the standard treatment of cryptococcosis remains insufficient, especially in resource-poor countries, where a high incidence of cryptococcal meningitis is observed^7^^-^^23^. In addition, increased fungal resistance to azoles, difficulty in administering and monitoring the adverse effects of amphotericin B and flucytosine, and their high costs remain important challenges in medical practice, even in resource-rich countries.

## THERAPEUTIC FAILURES

This phenomenon of antimicrobial resistance results in serious restrictions on the available options for cryptococcosis clinical treatment. Common antifungal resistance mechanisms include a decrease in the effective drug concentration, alterations or overexpression of drug targets, and metabolic deviations^45^. Thus, therapeutic failure in cryptococcosis may be related to both host factors and the existence of strains of *Cryptococcus* spp. that develop resistance to antifungal drugs^46^. 

Extrapolations from previous studies on other fungal species may improve our understanding of the resistance mechanisms employed by *C. neoformans*^7^ for which research is scarce. Reports of *Cryptococcus* spp. being resistant to amphotericin B are relatively rare; however, this phenomenon is already a concern^47^. The mechanisms that confer resistance to polyenes are related to mutations in ergosterol biosynthesis pathway genes, resulting in reduced binding of amphotericin B and/or inactivation of the drug, leading to fungal resistance^48^^-^^49^. The mechanisms of flucytosine resistance in *Cryptococcus* spp. remain unresolved and further investigation is needed to define them^7^. Approximately 10% of fungal isolates*,* even in the absence of previous drug exposure, show primary resistance to flucytosine^50^. In the case of infections with *C. neoformans* in particular, monotherapy with flucytosine is discouraged because of the rapid and frequent appearance of resistant isolates^51^. 

In the 1990s, especially in patients with HIV, the indiscriminate use of fluconazole resulted in the emergence of drug-resistant *Cryptococcus* spp. strains among susceptible populations^52^^-^^54^. Azole resistance is a relatively common event in recurrent episodes of cryptococcal meningitis^33^^-^^55^. The molecular basis of this resistance in *Cryptococcus* spp. is poorly resolved; however, overexpression of the AFR1 gene that codes for the azole efflux pump and point mutations in the ERG11 gene, that is, the gene encoding lanosterol 14α-demethylase as the target enzyme of azoles, have been associated with alterations in susceptibility to fluconazole in *C. neoformans*^7^^-^^56^^-^^59^. 

Resistance to fluconazole in *Cryptococcus* spp. may also be associated with heteroresistance, an adaptive mode of resistance against azoles^60^. This phenomenon refers to the heterogeneous susceptibility of a microorganism population to fluconazole, meaning that some clones are resistant whereas others are susceptible^61^. Resistant subpopulations gradually adapt to increasing drug concentrations. However, this acquired resistance to high concentrations of fluconazole can be lost during repeated passages in drug-free media and the clones return to their original level of heteroresistance^60^^-^^62^. 

The rise of heteroresistance in isolates of the *C. neoformans* species complex against fluconazole has been identified as one of the causes of cryptococcosis^63^. Heteroresistance may explain treatment failure in some patients, even when they are treated with the appropriate choices and concentrations of antifungal drugs^61^. Furthermore, current antifungal susceptibility testing algorithms have not been designed to detect heteroresistance; accordingly, unreliable susceptibility testing results are expected in the case of infections with heteroresistant *Cryptococcus* spp. strains^62^^-^^64^^-^^66^. 

## BIOPROSPECTING OF NATURAL PRODUCTS WITH ANTIFUNGAL ACTIVITY

Historically, nature has been an important source of therapeutic molecules. Currently, secondary metabolites of natural products produced by plants, microorganisms, marine animals, and other aquatic systems comprise approximately half of all pharmaceutical products on the market^67^^-^^68^. This reveals an immeasurable source of opportunities in the area of scientific and technological research on natural products, and prospecting new drugs from biodiversity remains one of the main choices for the identification of new drugs^69^^-^^70^. 

Bioprospecting of anticryptococcal drugs is commonly performed using classic or virtual (computational) cell screening*.* In the course of these screening approaches*,* bioproducts obtained from natural sources, such as plants, fungi, bacteria, insects, animals, and marine organisms^71^^-^^72^, were initially tested using bioassays that assess antifungal activity^10^. The disk diffusion assay is the most commonly used qualitative method for initial screening of antifungal activity^73^. The second most common method is the broth microdilution method, which is described by the Clinical and Laboratory Standards Institute (CLSI; document M-27 A4) or the European Committee on Antimicrobial Susceptibility Testing (document EDef 7.3.1), and is used to quantitatively determine the minimum inhibitory concentration (MIC) of substances with antimicrobial effects against pathogenic yeasts^74^^-^^75^. 

**FIGURE 2:**
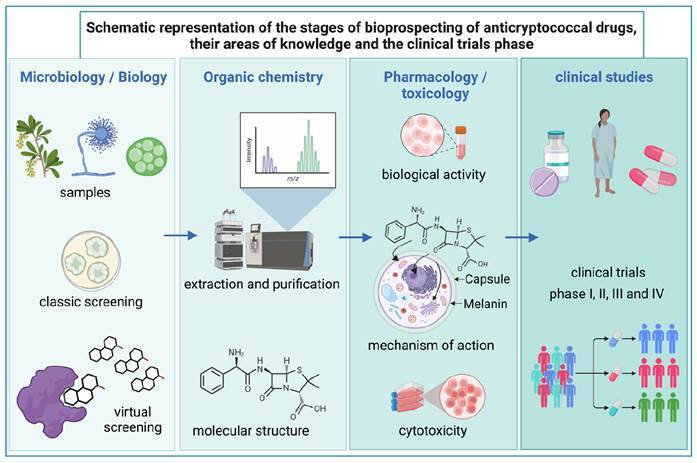
Bioprospecting steps for anticryptococcal drugs, their areas of knowledge, and the clinical trials phase. Created with BioRender.com.

Once the antifungal potential is identified, the bioproducts are subjected to extraction, isolation, and identification steps, which include different techniques capable of detecting the presence of compounds and then characterizing them^76^. In summary, the discovery of natural products with antifungal activity generally comprises: 1) classic or virtual cell screening; 2) extraction, isolation of compounds and structural characterization by thin layer chromatography, variations of chromatography associated with mass spectrometry, analysis of carbon 13 nuclear magnetic resonance, and hydrogen nuclear magnetic resonance analysis; 3) pharmacological studies to determine the mode of action; 4) toxicological studies to delineate the substance’s safety; 5) preclinical trials and, if successful; 6) clinical and marketing studies (Figure 2).

Several new natural products from fungi, bacteria, insects, sponges, algae, and plants have proven to be effective alternatives with the potential to form new drugs that can be effectively used against strains of *C. neoformans* and *gattii*^76^^-^^77^. In recent years, marine sponges and algae have emerged as important sources of new natural products with antifungal activity^78^; however, plants and fungi are still the most productive sources of antifungal compounds with anticryptococcal activity*,* including phenols, flavonoids, terpenoids, alkaloids, and peptides, as the main chemical classes represented in these plants^77^.

Natural products are important sources of therapeutic drugs. However, it is generally accepted that the drug discovery and development processes are time- and resource-intensive. Thus, in recent years, both computational and experimental techniques have played important roles and represent complementary approaches^76^. For a complete review of computer-aided drug design and virtual screening for lead molecules in the discovery of new drugs against *Cryptococcus* spp., the comprehensive work by Manjunath and Skariyachan (2018) should be consulted^79^. Table 1 summarizes the lead molecules selected from natural sources with antifungal activity against *Cryptococcus* spp. that have been identified in recent years.

**TABLE 1:** Lead molecules selected from natural sources with antifungal activity against *Cryptococcus* spp. that have been identified in recent years.

Source	Natural source	Compound/ chemical class	Reference
Plant	*Ocimum basilicum* (Linnaeus)	Sesquiterpenes	(80)
	*Lafoensia pacari* (St-Hilaire)	Punicalagin (tannins)	(81)
	*Thymus vulgaris* (Linnaeus)	Terpenoids	(82)
	*Xylosma prockia* (Turcz)	Phenolic metabolites	(83)
	*Uvaria comperei* (Le Thomas)	Alkaloid and flavonoids	(84)
	*Gentiana crassicaulis* (Duthie ex Burkill)	Bisphosphocholines	(85)
	*Chromolaena odorata* (Linnaeus)	Flavonoids	(86)
	*Cistus ladanifer* (Linnaeus)	Terpenoids	(87)
	*Hypoxis daylily* (Linnaeus)	Benzoylcyclopropane derivatives	(88)
	*Annona mucosa* (Jacquin)	Liriodenine	(89)
	*Verbesina turbacensis* (Kunth)	Hydroxycinnamic esters	(90)
Fungus	*Pestalotiopsis* sp.	Pestalactams	(91)
	*Auxarthron / Pseudauxarthron*	Phenalenones and cyclic tetrapeptides	(92)
	*Ruby discosia*	Chaetoglobosins	(93)
	*Preussia typharum*	Macrolides	(94)
	*Aspergillus terreus*	Terrestrial	(95)
	*Sodiomyces alkalinus*	Hydrophobins	(96)
Animal	*Hippospongia* sp.	Sesquiterpene quinones	(97)
	*Plakortis zyggompha* and *Plakortis halichondrioides*	Plakinic acid and plakortides	(98)
	*Tetrigone melanoleuca* and *Tetragonula laeviceps*	Propolis	(99)
	*Meccus pallidipennis* and *Rhodnius prolixus*	Peptides	(100)
Bacterium	*Streptomyces clavuligerus*	Ibomycin	(101)

## STRUCTURAL MODIFICATION

The first step in the design of new anticryptococcal drugs using structural modification is the use of a well-defined chemical substance with previously characterized biological activity^102^. The next step involves the techniques required to derive new analogs, homologues, or structural congeners with improved pharmacological properties. For this purpose, general processes of simplification and molecular association have been applied^102^^-^^104^. In summary, the final product was designed by the partial molecular modification of the prototype compound with the inclusion or exclusion of chemical structures that favor greater potency, stability, and safety characteristics than the original compound^68^. 

Substituted derivatives of terpenoids, quinones, naphthoquinones and coumaric acid are among the compounds with antifungal properties whose derivatives have been extensively studied in recent years for their anticryptococcal activity^105^^-^^110^. Recently, derivatives of sampagin, an alkaloid extracted from the stem bark of *Cananga odorata* Lamarck, have been shown to mediate potent antifungal activity against *C. neoformans* and *gattii* species^110^. In this study, a series of tricyclic isoxazole derivatives with excellent anticryptococcal activities were identified by structural simplification and alteration of the sample skeleton. The derived compound (Table 2) showed a high degree of inhibitory activity against *C. neoformans,* with an MIC_80_ value of 0.031 μg/mL. This activity was more potent than that of substances such as fluconazole and voriconazole. Furthermore, the substance showed potent inhibitory effects against important virulence factors, such as biofilm activity, melanin production, and urease activity of yeasts^110^. 

**TABLE 2:** Chemical structure of substituted derivatives with noteworthy activity against *Cryptococcus neoformans* and *Cryptococcus gattii* strains obtained by applying molecular modification.

Starting material (prototype)	Derivative with increased activity	Reference
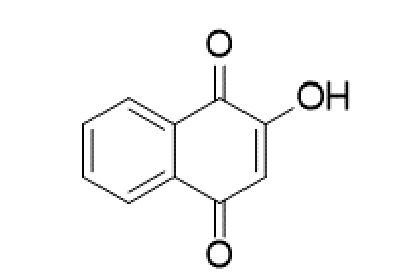	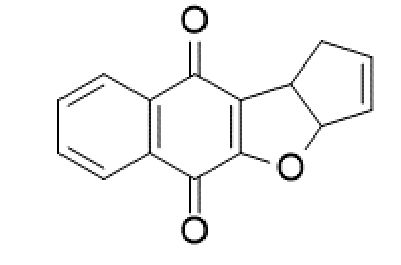	
		^109^
2-hydroxynaphthalene-1,4-dione	1 *H* -cyclopenta[ *b* ]naphtho[2,3- *d* ]furan-5,10(3a *H*,10b *H* )-dione	
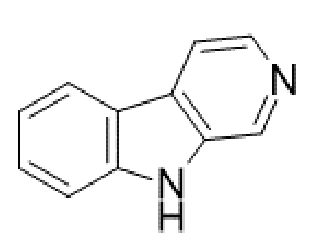	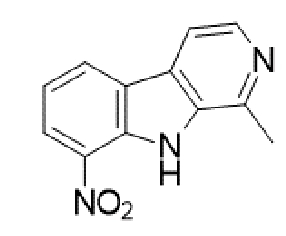	^108^
9 *H* -pyrido[3,4- *b* ]indole	1-methyl-8-nitro-9 *H* -pyrido[3,4- *b* ]indole	
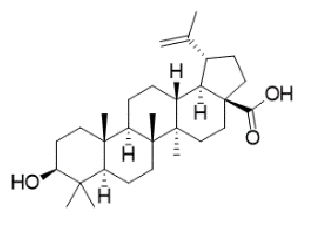	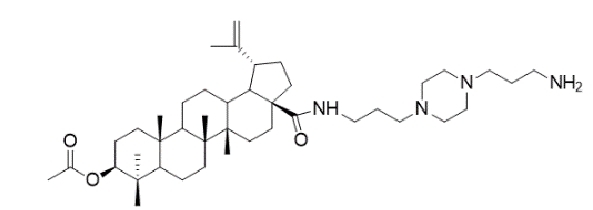	^111^
Betulinic acid	( *1R*,3a *S*,5a *S*,5b *R*,9 *S*,1(1a *R* )-3a-((3-(4-(3aminopropyl)piperazin-1-yl)propryl)carbamoyl)5a,5b,8,8, 11a-pentamethyl-1-(prop-1-em-2-yl)-icosahydro-1 *H* -cyclopenta[ *a* ]chrysen-9-yl acetate	
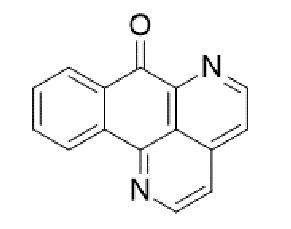	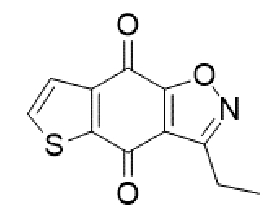	^110^
7 *H* -naphtho[1,2,3- *ij* ][2,7]naphthyridin-7-one	3-ethylthieno[3',2':4,5]benzo[1,2 - *d* ]isoxazole-4,8-dione	
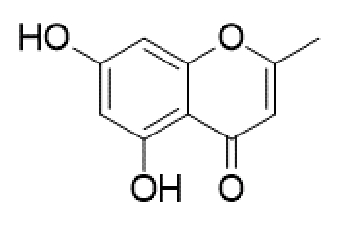	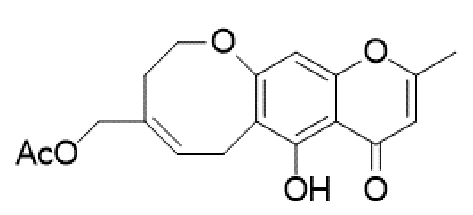	^112^
5,7-dihydroxy-2-methyl-4 *H* -chromen-4-one	( *E* )-2-(5-hydroxy-2-methyl-4-methylene-4,6,9,10-tetrahydrooxocino[3,2- *g* ]chromen-8-yl)ethyl acetate	
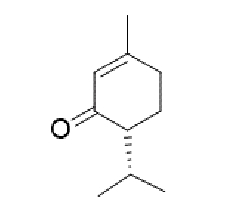	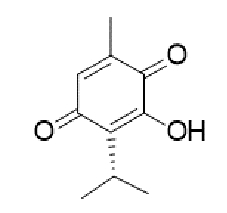	^113^
( *S* )-6-isopeopyl-3-methylcyclohex-2-enone	3-hydroxy-2-isopropyl-5-methylcyclohexa-2,5,-diene-1,4-dione	
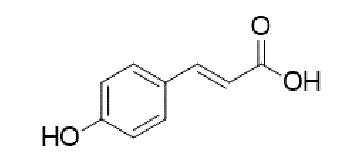	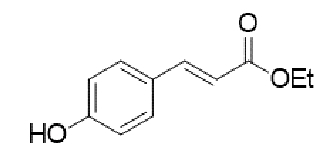	^114^
( *E* )-3-(4-hydroxyphenyl)acrylic acid	( *E* )-ethyl 3-(4-hydroxyphenyl)acrylate	
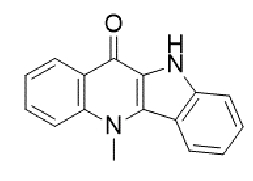	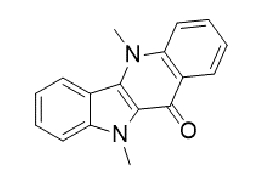	^105^
5-methyl-5 *H* -indolo[3,2- *b* ]quinolin-11(10 *H* )-one	5,10-dimethyl-5 *H* -indolo[3,2- *b* ]quinolin-11(10 *H* )-one	

Structures were designed using Chemdraw 19.0

Despite the considerable efforts invested in the search for antifungals, several new compounds that were screened or obtained by structural modification and demonstrated antifungal activity against *Cryptococcus* spp. remain poorly investigated^77^. However, there is hope that some will progress into useful antifungal agents owing to molecular modifications. Moreover, in the next step, such new drugs with anticryptococcal activity will hopefully advance to clinical trials.

## DRUG REPURPOSING

To accelerate the development of new antifungal agents, drugs developed for other therapeutic purposes can be repurposed if they also show antifungal activity^2^. Wemuth was an early advocate of screening approved drugs for new therapeutic indications and coined the term systematic optimization of side-activities (SOSA), which has become well known as a drug repositioning strategy^115^. 

The repositioning of drugs has few advantages, namely: 1) pharmacological, pharmacokinetic and safety data in humans have already been previously established in preclinical and human trials, 2) the clinical use of a drug already available on the market is immediate, and 3) reduction in research costs associated with the expansion of the therapeutic indication^8^^-^^115^^-^^116^. Therefore, expanding the applicability of a drug to other diseases is a promising approach that has been successfully used in recent years to identify drugs with antifungal activity^37^. 

In recent years, a series of drugs developed for other therapeutic purposes have demonstrated antifungal activity against *Cryptococcus* spp.^117^^-^^130^. The most notable examples of repurposed pharmaceutical compounds for cryptococcal meningitis that have reached the clinical trial stage involve the drugs sertraline and tamoxifen^2^^-^^117^. Tamoxifen has not shown any benefit in eliminating *Cryptococcus* spp. from the cerebrospinal fluid, and the sertraline study had to be terminated early due to serious adverse effects^116^^-^^117^. It is important to note that repurposed drugs are not optimized for indications other than those on the leaflet. Therefore, their pharmacokinetic properties and efficacy often need to be improved if off-label applications are desired. Considering this observation, an alternative approach to repurposing is the optimization of a compound or drug for its secondary effect, also known as SOSA^115^. For a comprehensive review of this approach, please refer to the recent work of Donlin and Meyers (2022)^118^. 

## COMBINATION THERAPY

Compared with the discovery of antibiotics, the discovery of antifungal agents is much more difficult. A common explanation for this finding is that fungus, similar to its human host, is a eukaryotic organism. This phylogenetic relatedness hinders the development of effective antifungal agents that are nontoxic to human cells^130^. This problem is evident within the *Cryptococcus* genus because of the pathogenicity, virulence, and resistance mechanisms that these fungi have developed^6^. In this context, combining different drugs for antifungal therapy is a feasible strategy to increase the efficacy of antifungals, decrease and/or avoid toxicity, and prevent fungal resistance.

The commonly used mode of assessing the combined effects of the two substances is the checkerboard test^131^^-^^133^. This method is based on the broth microdilution technique, in line with document M7-A4 of the CLSI^74^. Table 3 summarizes published drug combination studies of amphotericin B and fluconazole against *Cryptococcus* spp. In summary, the presented combinations are associated with improved activity of conventional antifungal agents owing to the combined action of more than one target, as well as reduced toxicity, because small amounts of one or both drugs can be used in combination^12^. An example of this is flucytosine, which seems to be of little use when used on its own for cryptococcosis therapy but has been reported to act synergistically in combination with amphotericin B. Therefore, additional benefits for the treatment of cryptococcal meningitis are observed when this drug is used in combination^8^. Consequently, combined antifungal therapy using flucytosine and amphotericin B has been used for at least four decades. However, as mentioned previously, the adverse effects, high cost, and unavailability of flucytosine in resource-poor countries still negatively interfere with the treatment of cryptococcal meningitis^25^^-^^39^. 

**TABLE 3:** Studies assessing combinations of drugs or bioactive compounds with promising antifungal activity against *Cryptococcus* spp.

Combination	Screening	Reference
**Coumaric acid analogues + amphotericin B**	Checkerboard assays	^114^
**Artovastatin + fluconazole**	Checkerboard assays	^120^
**Curcumin + amphotericin B**	Checkerboard assays	^134^
**Dicyclomine + fluconazole**	Virtual library	^135^
**Duloxetine + fluconazole**	Checkerboard assays	^136^
**Erythromycin + amphotericin B**	Virtual library	^37^
**Fluoxetine + amphotericin B**	Checkerboard assays	^137^
***Glimepiride*+ amphotericin B**	Virtual library	^37^
**Lactoferrin + amphotericin B**	Checkerboard assays	^138^
**Minocycline + fluconazole**	Checkerboard assays	^10^
**N-acetylcysteine + amphotericin B**	Checkerboard assays	^139^
**Simvastatin + amphotericin B**	Checkerboard assays	^140^
**Tamoxifen + amphotericin B**	Checkerboard assays	^117^
**Triclosan + fluconazole**	Checkerboard assays	^141^
**Pedalitin + amphotericin B**	Checkerboard assays	^142^
**α -Cyperone + fluconazole**	Checkerboard assays	^143^

There is some hope on the horizon, with the new antifungals fosmanogepix and opelconazole, which are in the advanced stages of clinical development and exhibit antifungal activity against *Cryptococcus* spp. However, the available antifungal therapies for this infection remain limited. The adverse effects and high costs of the combined amphotericin B and flucytosine therapy, as well as the emerging resistance of *C. neoformans* and *C. gattii* to fluconazole, pose considerable challenges to clinical treatment. To overcome these problems, the use of drugs and combination therapies has attracted considerable attention in recent years. These methodologies have been increasingly applied because they are associated with a fast and economical mode of searching for new antifungal agents with antifungal activity against cryptococci. In parallel, research on the bioprospecting of natural products and studies, including planned structural modifications of bioactive molecules, continues in research laboratories. These combined efforts have fueled the ongoing hope of identifying a successful new antifungal agent, either by screening or targeted modifications of pre-existing molecules.
